# The lure of decentralized social media: Extending the UTAUT model for understanding users’ adoption of blockchain-based social media

**DOI:** 10.1371/journal.pone.0308458

**Published:** 2024-08-07

**Authors:** Anatoliy Gruzd, Alyssa Saiphoo, Philip Mai

**Affiliations:** Ted Rogers School of Management, Toronto Metropolitan University, Toronto, ON, Canada; University of Pisa, ITALY

## Abstract

The study uses 31 semi-structured interviews to explore users’ motivations for adopting and using blockchain-based social media (BSM) platforms. The objective of the study is twofold—to collect empirical data on early adopters of BSM and to test the applicability of the Unified Theory of Acceptance and Use of Technology (UTAUT) model for explaining why some users are choosing BSM over mainstream social media (MSM) platforms. Manual content analysis of the interviews reveals that users are initially drawn to BSM due to social influence and financial incentives, but they continue to use it mainly because of the sense of community they experience. We also find that the steep learning curve, the absence of content moderation, as well as security and privacy concerns hinder the widespread adoption of these platforms. From the theoretical side, although the UTAUT model is generally suitable for examining why individuals use BSM, we suggest integrating two additional factors into the model: financial incentives and content moderation.

## Introduction

Mainstream social media platforms (MSM) such as Facebook, YouTube, and TikTok have become a ubiquitous part of modern life, regularly used to connect with friends and family [1], follow influencers and celebrities [2], access and share news [3], and gather information about products and services [4]. Despite the benefits of MSM, some users are turning to blockchain-based social media platforms (BSM) due to concerns over privacy, misinformation, and enforcement of content moderation policies by social media companies. Given the novelty of BSM, there is a knowledge gap regarding their effectiveness, best practices, technological and management needs, variations across settings, and broader impacts on society. This study explores the adoption and use of BSM to understand their impact on the social media landscape. In this paper, we will use the term BSM to refer to blockchain-based social media while acknowledging alternative terminology used in the literature [5, 6], such as Blockchain Social Networks (BSN), Blockchain Online Social Media (BOSM), and Distributed/Decentralized Online Social Networks (DOSN).

Blockchain technologies are often associated with sectors such as finance, healthcare, real estate, and supply chain management [7]; however, the same technology that gave rise to cryptocurrencies [8] and non-fungible tokens [9] can also support communication exchange between users, including the publication of user-generated content. Since BSM platforms are built on top of a blockchain, they inherit several properties from blockchains regarding how user data is stored and shared. First, BSM users’ data is kept across a network of nodes instead of a central server [10]. Since the data is decentralized, there is no overarching governing body to decide what can or cannot be posted by its users [11]. Second, BSM users’ data cannot be easily deleted or altered [12]. Third, transactions on a blockchain require multi-signature protection, making it difficult for malicious users to conduct fraudulent transactions. Another feature setting BSM platforms apart from MSM is that they offer incentives to their users through cryptocurrency for actions and activities typical of a social media platform, such as posting content or engaging with content posted by others. Unlike MSM, BSM platforms allow anyone to create a new front-end or functionality through decentralized applications (dApps) that run on a blockchain as opposed to applications designed for a single computer [13].

In this study, we focused on the following three blockchain-based platforms that were active during our research and were frequently mentioned by our study participants: Steemit, Hive, and Minds. Steemit was the first widely adopted BSM platform, operating on the Steem blockchain and designed to support decentralized social applications [14, 15]. Steemit users can join communities and create blog posts, receiving platform-specific cryptocurrency as a reward for content creation or engagement with other accounts on the platform. In 2019, due to concerns over the centralization of the platform after its acquisition by the Tron Foundation, the Steem community created a copy of the Steem blockchain, called the HIVE blockchain [16], transferring all content and tokens to the new network. As of 2023, the HIVE blockchain consists of an ecosystem of various dApps, including social blogging interfaces such as Hive.blog and PEAKD, video sharing platforms like 3Speak, and online games like Splinterlands. Another popular BSM platform is Minds, which runs on the Ethereum blockchain network. Minds is a microblogging platform that rewards users with Ether, a form of cryptocurrency, for various actions, such as receiving upvotes, comments, and subscribers. Its creation was inspired mainly by content moderation issues on Twitter rebranded as X in 2023 [12].

Our study is motivated by the increasing number of privacy and data protection regulations being introduced globally, which typically do not account for decentralized applications like BSM. For instance, regulations like the General Data Protection Regulation (GDPR) assume that a single entity oversees the collection of personal information. However, this concept is challenged by decentralized blockchain networks, which distribute data across multiple nodes and lack a central authority. The tamper-proof nature of blockchains also conflicts with the “right to be forgotten” provision of GDPR and similar regulations, which allow users to request the deletion of their personal information. Understanding how BSM platforms work, who joins them, and why, can inform regulators and other stakeholders about the shifting power dynamics between data controllers and producers through new ways of creating, collecting, accessing, sharing, and rewarding user data.

To shed light on the adoption and use of BSM, we conducted semi-structured interviews with a sample of BSM users, whom we view as early adopters. Through these interviews, we aimed to gain insights into their perspectives and answer the following research questions:

- RQ 1: What motivated early adopters to use blockchain-based social media platforms?
- RQ 2: What factors contribute to the continued use of blockchain-based social media platforms?
- RQ 3: What are the main barriers to adopting and using blockchain-based social media platforms?
- RQ 4: How does using cryptocurrencies as incentives for content creation and engagement affect the behavior and perception of social media usage?
- RQ 5: How has adopting blockchain-based social media platforms influenced individuals’ usage habits and behaviors on mainstream social media platforms?

The rest of the paper is structured as follows: The Literature Review section provides an overview of BSM-related studies, the UTAUT model, and its application to the adoption of blockchain technology. The Method section details the participants’ recruitment and interview process, as well as the transcription and analysis of the collected data. The Results section presents the findings from the perspective of the UTAUT model. In the Discussion section, we revisit the research questions and examine the potential expansion of the UTAUT model to enhance future studies on blockchain technology. The Conclusion highlights key outcomes and suggests avenues for further research in blockchain technology adoption.

## Literature review

### Social media meets blockchain

Based on a systematic literature review conducted between 2017 and 2022 [17], only 42 academic articles (in English) examined the application of blockchain technology in social networking. Even fewer have studied how and why individuals adopt and use BSM. Most of this early work focused on proposing a proof of concept or discussed technical aspects of implementing BSM [18], with a particular emphasis on developing prototype systems to reduce the spread of misinformation [19–21]. Some also proposed various user privacy and data protection models for social networking [11, 22, 23]. In this section, we will review studies on the uses and users of BSM. We start with a brief overview of the Steem blockchain and Steemit (a BSM application operating on the Steem network) due to their prevalence in prior research. Since this is an emerging area with a continuously increasing body of literature [e.g., 24–26], this section is not intended to be a systematic literature review.

Like any blockchain network, Steem has a cryptocurrency called STEEM to facilitate transactions and transfers. STEEM is often characterized as ‘gold’ because it is created in the ‘mining’ process. Unlike the Bitcoin blockchain, where anyone with proper computing power can become a miner, Steem is a so-called delegated proof-of-stake (DPoS) network; that is, only a limited number of miners (known as ‘witnesses’) voted in by the community of users are ‘delegated’ to create blocks and in return are rewarded with cryptocurrency for doing so [27]. The DPoS model speeds up the creation of new blocks, reducing costs and environmental impact associated with their creation; however, it also makes the network more centralized and vulnerable to influence by accounts with significant shares in the network [28].

The Steem network has two additional digital assets tied to STEEM: Steem Blockchain Dollar (SBD) and Steem Power (SP). SBD is a stable-value token with a 1:1 exchange rate to the US dollar. Unlike SBD, SP is not a fungible asset and cannot be traded directly for other currencies, but it can be converted into STEEM and vice versa in the process called Powering Up (STEEM to SP) or Powering Down (SP to STEEM). SP plays an essential role in the management of the network. It is used to reward witnesses for creating blocks, and content creators and curators (those who upvote or reshare others’ posts). The amount of SP a user holds relates to their reputation on Steemit and influences the payout content creators and curators receive for popular content. While any Steem user can vote for a witness, votes by users with more SP weigh more.

In one of the first studies on Steemit, Thelwall [29] analyzed nearly 1M original posts in English shared between March 2016 and July 2017. The study found that the initial post, on average, received 1.22 cents; however, most posts (82%) did not receive any reward due to the lack of engagement from other users. The author suggests that, given the relatively low payouts per post, financial incentives might not serve as the primary driver for participation on Steemit. At the same time, since some posts did receive substantial payouts, a ‘lottery-like’ incentive structure may still exist. Unfortunately, the study does not provide additional insights into the underlying reasons since it did not examine user perceptions.

Liu et al. [5] studied the factors influencing users’ participation in Steemit. The authors analyzed a dataset containing information about posts, profiles, and engagement data for around 25k users who were active on the platform between March 4 and May 20, 2019. Their analysis revealed that social and economic motivations likely drive engagement on the platform. Social motivations were operationalized through metrics such as the number of followers and friends (users they follow). Economic factors were assessed based on the amount and percentage of SP held by users. While not tested directly in the study, the authors posit that users’ earning and holding SP may make them feel a stronger sense of community and investment in the platform.

Guidi et al. [30] also examined the role of social and economic factors in driving user engagement based on over 22M public transactions in the Steem blockchain between 2017 and 2018. The study found that the Steem network had 1.24M accounts during the studied period, with about 37% exhibiting no interactions with other users. The most common types of interactions included: (a) upvoting or downvoting someone’s content, (b) following another user or resharing their content, and (c) commenting on someone’s post. Financial transactions ranked only as the fourth most frequent transaction type, including actions like transferring or trading cryptocurrency with others. Interestingly, the study identified bots as some of the most active accounts on the network, whose primary role was to amplify users’ posts for a fee. Li and Palanisamy [28] analyzed over 500M transactions by around 1M users from March 2016 (when Steemit was launched) to August 2018, finding that approximately 16% of all transactions were for amplification services when a bot-suspected account upvoted user-provided posts within 7 days of receiving the payment. Furthermore, authors of most popular posts often contacted multiple bot-like accounts to promote their posts. To combat such activities, Steemit introduced a reward policy change by reducing the share of tokens allocated to the original poster (from 75% to 50% of the total reward) to discourage these practices. However, Delkhosh et al. [31] found that this policy backfired since the change reduced the number of original posts as the authors were less incentivized to contribute.

Another area of research focused on the content shared by users on Steemit to gain insights into the topics they are interested in and how those interests may have evolved. Kapanova et al. [32] examined around 80k English posts shared on Steemit between January 2017 and September 2019. The results indicated that domain-specific posts about cryptocurrencies and blockchains have become more prevalent. This is likely due to new users joining the platform who are interested in learning how cryptocurrencies and the platform work. In related work, Guidi et al. [33] examined what topics witnesses share on Steemit. The role of a witness is highly sought-after since witnesses are rewarded with cryptocurrency for producing new blocks for the Steem network. The researchers reviewed the posting behavior of 100 highly ranked witnesses, finding that they post on various topics, including procedural ones related to block creation. Despite their vital role in the network, witnesses tended not to gain a high reputation for their content. For example, only 10% of witnesses got over 10,000 followers on the platform with over 1M accounts. The paper also documented a shift in how witnesses engage on the platform, from posting original content and reposting others at least once daily to a much lower engagement rate, likely due to diminishing interest in Steemit in 2020 overall. The findings emphasize the need for future research involving users to offer a more comprehensive rationale for these trends.

The final line of research examined how BSM users migrate from one platform to another. A distinctive feature of BSM is the users’ ability to initiate a new version of the network. Minor adjustments to the platform’s functionalities are called ‘soft forks’ as they maintain backward compatibility with the previous versions of the blockchain network. However, when more substantial changes are introduced, they are called ‘hard forks.’ Users can stay or migrate to the new network when a hard fork is created. Ba et al. [34] examined the migration process of users to Hive, a hard fork of Steem, in March 2020. The reason for the hard fork creation by the community was in response to the takeover by a private entity (Tron Foundation) that acquired enough tokens to select their preferred witnesses and limit the action the rest of the community could take. At the time of the fork, the new network had identical accounts and assets, making it easier for users to switch to the new platform. The study collected all transactions related to social interactions and economic-related operations for the two networks from their launch (March 2016 for Steem; March 2020 for Hive) until January 2021. The results suggest that while the two networks started with the same set of accounts, the overlap in the number of active users on both platforms has decreased, indicating that each platform has established its user base.

Extending previous research that mainly examined macro dynamics through observed transactions, our approach seeks to provide a more nuanced view of BSM from the user perspective. We aim to offer a qualitative analysis of the user experience with BSM, focusing on factors that influence its adoption and continued use. Informed by the literature, we will consider the role of key actors, such as witnesses and bots, as well as potential issues related to migrations between BSM platforms.

### Unified theory of acceptance and use of technology model

The UTAUT is a widely cited model in technology adoption and usage [35]. This model combines several key theories, including the Diffusion of Innovation Theory by Rogers [36] and the Technology Acceptance Model by Davis [37]. The UTAUT model provides a comprehensive framework for understanding technology adoption and usage behaviors by examining and considering several factors. The original UTAUT model included four factors [35]: performance expectancy, effort expectancy, social influence, and facilitating conditions. Performance expectancy refers to the degree to which the technology benefits the user. Effort expectancy refers to the ease of using the technology. Social influence refers to the impact of the technology employed by influential others on an individual’s decision to adopt and use a particular technology. Facilitating conditions refer to the availability of resources and support for using the technology. Later, the UTAUT model was expanded to include three additional factors [38]: hedonic motivation (the enjoyment of using the technology), price value (the balance of benefits and costs), and habit (the passage of time from initial use).

The UTAUT model has been previously applied to explain the adoption and use of blockchain technology across various industries and sectors in society. In the financial sector, social influence was identified as a significant factor in accountants’ intention to adopt blockchain technology [39]. Performance expectancy and facilitating conditions were positively related to the behavioral intention of Indian bankers to use blockchain technology [40], and enabling conditions and social influence were key factors in consumers’ intention to use blockchain-based digital banking [41]. The model has also been applied to the supply chain industry, where performance expectancy and facilitating conditions have been found to play a significant role in adopting blockchain technology [42, 43].

Our study extends this line of research and examines the applicability of the core factors in UTAUT for understanding individuals’ decisions to adopt and use BSM platforms. We discuss the role of two UTAUT factors that have yet to be reviewed in blockchain technology adoption: hedonic motivation and habit. Published studies on the adoption and use of blockchain technology have not fully examined the role of hedonic motivation, which has shown to be a crucial factor in the adoption and use of MSM platforms [44]. Habit is another UTAUT factor not fully explored in previous blockchain studies. We believe these two UTAUT factors may play a role in differentiating usage patterns and motivations between early and mature BSM users. The price value factor will not be included in our examination as BSM platforms are typically free to use (for those with access to an internet-accessible device such as a smartphone or computer), rendering this factor inapplicable in this research context.

### Additional factors influencing adoption and use: Risk and trust

Several UTAUT-driven studies proposed to expand the model by including risk and trust as additional factors. Risk hinders technology adoption and refers to users’ feelings of anxiety or uncertainty towards the technology and the potential for negative consequences that may result from its use [45]. Despite the potential benefits associated with using BSM platforms, users may perceive a high risk associated with this new technology, making them less likely to adopt it [46, 47].

On the other hand, trust has been shown to impact the behavioral intention to adopt technology [48] and has also been proposed as an extension to the UTAUT model [49, 50]. Trust refers to the belief that an exchange or interaction will fulfill obligations [51]. Although blockchain technology is often described as “trustless,” meaning data consensus is reached through code instead of relying on a single party [52], trust is still necessary for the front-end of BSM platforms, which depends on the actions of other users [53]. As a result, trust is likely to be a significant factor in the adoption and use of BSM platforms.

### BSM-specific factors: Financial incentives and content moderation

The monetization of content and engagement with cryptocurrency is a feature of BSM platforms that may influence adoption and use. Thus, we anticipate identifying additional UTAUT factors related to financial incentivization through cryptocurrency.

Additionally, the lack of content moderation on BSM platforms may play a significant role in the adoption and use, particularly among individuals dissatisfied with the moderation policies of MSM platforms. Users who have been banned from Facebook, YouTube, or another platform for violating the platform’s community standards may see the absence of content moderation as a compelling factor when considering BSM platforms.

## Method

To understand the motivations and experiences of users of popular BSM platforms (Steemit, Hive, and Minds), we conducted semi-structured interviews with users via the Zoom virtual meeting platform. Our goal was to uncover the factors that drive their adoption and use of BSM platforms. The transcripts of the interviews were analyzed by two coders (two of the authors) to identify relevant factors, including those predicted by the UTAUT model. This allowed us to critically evaluate the interviews and determine the suitability of the UTAUT model in studying BSM platform adoption and usage.

### Data collection

After receiving approval from the University Research Ethics Board, study participants were recruited through several channels. First, we recruited participants directly through Steemit, Hive, and Minds by posting an open call for participants using a designated research account. Second, we employed direct messaging on Twitter to reach potential participants who promoted BSM among Twitter users. To identify potential participants, we searched for public posts mentioning one of the BSM-related hashtags: #hive, #steemit, and #minds. Third, we relied on snowball sampling for recruitment, in which participants were given a study-designated email address to share with other users of BSM platforms where they could contact us if interested in participating.

Once participants expressed interest, we directed them to the dedicated study page that included the main points of the consent form, details about the interview process, and a link to the pre-screen online form. The pre-screen form was designed to confirm their eligibility for the study and collect consents. To be eligible, participants had to be: (a) 18 years old or older, (b) active users of at least one BSM platform (we defined “active” user as a person who signs in to one of these platforms at least once weekly and posts at least once monthly), and (c) able to understand and speak English fluently.

Once participant eligibility was determined and their consent form was received, participants were assigned a unique study ID number and were directed to an online scheduling website where they could sign up for an interview slot. Interviews were conducted from June 16th to August 11th, 2022, by a single interviewer and consisted of 13 open-ended questions. Verbal consent was reaffirmed before the start of the interview.

Participants were asked how they found out about BSM platforms, which ones they currently use, and their use of MSM platforms. Questions can be found in S1 Appendix. Interviews were semi-structured; these questions were used as a guide, but the interviewer prompted participants on relevant topics as they came up.

Thirty-two virtual interviews were conducted. One participant did not provide consent for the audio recording of their interview, and as a result, their interview was not included in the content analysis. This resulted in a final sample size of 31 participants, which was deemed to be an adequate number of interviews to reach a point of saturation; a review of prior empirical studies by Hennink and Kaiser [54] found that a sample size of 9–17 interviews is sufficient to reach saturation in qualitative research. The interviewer took extensive notes during the interview that was not recorded, capturing the key points of the conversation. Later, these notes were compared to the coded results of the transcribed interviews. The comparison confirmed that the recorded interviews had already captured the key themes from the non-recorded interview.

The sample of 31 participants predominantly self-identified as men (77% men, 23% women). The largest age group represented was 25–34 (52%). Nearly half of the participants come from Nigeria (48%), followed by the United States (16%). A bachelor’s degree was the most common highest level of education held by participants (48%). Self-employment was the most prevalent employment status (39%), followed by unemployment and seeking work (23%). See Table 1 for details.

**Table 1:** User demographics.

Demographic Group	Total	%
All Participants	31	100%
**Gender Self-Identification**		
Men	24	77%
Women	7	23%
**Age Group**		
25–34	16	52%
35–44	7	23%
45–54	4	13%
18–24	2	6%
55+	2	6%
**Country**		
Nigeria	15	48%
United States of America	5	16%
Mexico	2	6%
Venezuela	2	6%
Bulgaria	1	3%
United Kingdom	1	3%
Philippines	1	3%
Canada	1	3%
France	1	3%
Greece	1	3%
Not provided	1	3%
**Highest Level of Education**		
Bachelor’s degree	15	48%
Some college, no degree	5	16%
Master’s degree	5	16%
College diploma	5	16%
Doctorate degree	1	3%
**Employment Status**		
Self-employed	12	39%
Unemployed and currently looking for work	7	23%
Employed full time	6	19%
Employed part time	4	13%
Retired	1	3%
Homemaker	1	3%

The semi-structured interviews lasted between 17 and 70 minutes, with an average duration of 42 minutes. The audio was recorded using the Zoom recording feature, and subsequently, an automated transcript was generated. The interviewer and two research assistants manually reviewed and corrected these transcripts to ensure accuracy.

To protect the confidentiality and anonymity of participants, the following measures were implemented: participants’ true identities were known only to the research team, randomly assigned numbers were used instead of names in all transcriptions, and any identifying characteristics or references were removed from public reports and publications.

### Data analysis

Independent coding of transcripts was carried out by two authors using NVivo (ver. 1.7) over six months. The analysis was guided by the original UTAUT and common extensions to the model, as discussed in the Literature Review.

A thematic coding scheme was developed, with additional subcodes added under each factor based on themes that emerged during the content analysis. The coding was performed at the sentence level, and in cases where a statement referenced more than one factor, double coding was allowed. This approach to coding was implemented to capture the dual nature of some of the factors raised by the participants, allowing for a more nuanced understanding of the data.

The coding process employed an iterative approach. Each coder would code transcripts independently, and their coding was compared using NVivo. Any disagreements were resolved by reviewing each other’s coding and reasoning, first asynchronously and then synchronously. The level of agreement between coders was high, with Cohen’s paragraph-level overall kappa being 0.76 and Cohen’s sentence-level overall kappa—0.71 (see Table 2). Updates were made to the codebook and definitions throughout the process, as needed. The final version of the codebook can be found in S2 Appendix.

**Table 2:** Top-level codes.

Codes	Aggregate number of items coded	Aggregate number of coding references	Inter-rate reliability (Cohen’s kappa–paragraph level)	Inter-rate reliability (Cohen’s kappa–sentence level)
**UTAUT factors**				
Hedonic Motivation	31	648	0.75	0.70
Facilitating Conditions	31	368	0.77	0.68
Effort Expectancy	30	326	0.80	0.74
Performance Expectancy	27	221	0.69	0.72
Social Influence	27	123	0.79	0.66
Habit	27	105	0.73	0.75
Price Value	N/A	N/A	N/A	N/A
**UTAUT extension factors**				
Risk	27	168	0.66	0.65
Trust	22	134	0.71	0.61
**New factors**				
Financial Incentives	31	362	0.82	0.73
Content Moderation	24	248	0.76	0.68

## Results

### Coding overview

We found that the adoption and use patterns of the study participants on BSM align with the UTAUT model and its common extensions. The top-level codes identified in our study are listed in Table 2 below.

The most frequently referenced factor was hedonic motivation, with all participants expressing their enjoyment of using BSM platforms, particularly when interacting with other community members.

Facilitating conditions, the second most frequently mentioned factor, was also referenced by all 31 participants but reflected both positive and negative experiences with BSM. On the one hand, many participants liked the interface affordances offered by BSM, such as the ability to access data stored on the same blockchain through different dApps. On the other hand, several raised concerns about the lack of formal documentation or support options.

Effort expectancy was mentioned by 30 participants and overwhelmingly referred to as a barrier to the adoption and use of BSM. Participants noted the increased effort required to use BSM, especially during the onboarding process.

Performance expectancy was referenced by 27 participants when describing a perception of increased usefulness and efficiency of BSM over MSM for specific tasks, such as running crowdsourcing initiatives or reaching a more focused audience.

Social influence was mentioned as the main factor when learning about BSM, with 27 participants stating that they started using BSM due to a recommendation from someone in their circle or an online influencer.

Habit was referenced by 27 participants as a reason for their continued use of one or more BSM platforms.

The risks associated with using BSM were discussed by 27 participants, including potential scams, privacy and security breaches, and the possibility of a BSM platform going out of business. Despite these risks, 22 participants expressed trust in BSM (developers, other users, or smart contracts).

In addition to the factors predicted by the UTAUT model, our study also revealed several other factors influencing the adoption and use of BSM. Financial incentives, such as the possibility of earning reward tokens (cryptocurrency), were mentioned by all participants as a significant driver of their decision to join a BSM platform. Content moderation was seen as a positive aspect by 24 participants, encouraged by a lack of censorship and open access to the platform.

### In-depth analysis

Below is a more in-depth description of each code and subcode that emerged from our analysis of the transcripts. The results are presented in order of their prevalence in data.

#### Hedonic motivation

The code **hedonic motivation** was used to discuss different aspects of BSM platforms that the participant perceived to be enjoyable. Overall, this code was used 648 times by all 31 participants (see Table 3).

**Table 3:** Subcodes under hedonic motivation.

Code > Subcode	Number of Items Coded	Number of Coding References	Percentage of Participants That Used
Hedonic Motivation	31	648	100%
> Sense of Community	30	274	97%
> Usage Enjoyment	29	97	94%
> Learning and Teaching	25	138	81%
> Growing the Community	19	62	61%
> Novelty Enjoyment	14	39	45%
> Crowdfunding and Charity	11	37	35%

The most frequently used subcode in our analysis was **sense of community**, which was applied to statements expressing enjoyment of the platform due to the sense of belonging to one or more communities on the platform. In identifying text to be classified under this subcode, we looked for indications of various aspects commonly associated with being part of an online community, including feelings of belonging, recognition by others in the community, sustained membership over time, and the ability to influence others [55–58]. The analysis revealed that most participants experienced the sense of community. Specifically, they highlighted the pleasure they derived from interacting with like-minded individuals. A subset of participants noted a particular affinity for interactions with individuals from their own country, while others expressed a preference for connecting with individuals from other countries, as demonstrated in the following quote:

“Yeah, getting to meet new people, especially not [from] my country alone, outside, and I get to see how they do things; their culture, what their reason, how they view—[those are] just basically the things that got me attracted to it.”

Related to the sense of community was the subcode **growing the community,** which appeared when discussing how participants used online or offline channels to get more people using BSM. The idea was often that if it helps the platform, it will also help the participants. For example, one participant said:

“One of the reasons is that by helping Hive, I’m helping myself. By bringing in new people, those new people that I grow in the blockchain, they are going to be following me, so I increase my networking as well.”

Another subcode which is broadly related to the sense of community was **crowdfunding and charity**. It was used when participants discussed the enjoyment they experienced from being involved in crowdfunding, crowdsourcing, citizen science, or charity opportunities through the platform. This often involved the participant perceiving these activities as enjoyable not for their direct benefit but rather for the benefit of others.

The second most-used subcode in this broad category was **usage enjoyment**. It was used when participants talked about their joy of using the platform or that the use of the platform is perceived to be fun without directly referencing the community aspects of being on the platform. For example, a participant said: “And I tried, it was fun, I got nice reactions, expressions, nice interactions. So, I said, okay fine, let’s keep it”. A related subcode was **novelty enjoyment**. This code was also used for discussions of the participant perceiving the use of the platform as fun, interesting, or novel in some way. An example would be a participant saying that “I think it’s a good thing to experience. That’s new, and it’s always good to experience new possibilities.”

The third most-used subcode under the category of hedonic motivation was **learning and teaching**, which was used when participants expressed their enjoyment of using the BSM platform to learn from or instruct others. For example, one participant noted: “I post content, then I also read other people’s contents to learn more about what’s happening all over the world, and even in the blockchain space.” In analyzing statements pertaining to this subcode, we employed the Learning in the Wild coding schema, a framework designed to examine online learning and participation within online communities [59, 60]. This schema encompasses a variety of codes that aid in identifying and analyzing different elements of online learning, as evidenced through the content analysis of text-based asynchronous interactions. Although this framework was initially developed and validated in the context of MSM platforms such as Reddit, it applies to other online communities, including those on BSM platforms.

#### Facilitating conditions

The code **facilitating conditions** was used in instances where the participant was discussing the technical or organization infrastructure that exists or does not exist to support the use of a BSM platform. This code was used by 31 participants, meaning that it was a contributing factor for the adoption or use of BSM platforms for all participants. Below is a detailed review of several subcodes used to flag specific factors commonly mentioned. While some factors were noted in a positive light, others were raised as something that was hindering adoption or use. They are indicated with the plus or minus sign correspondingly in Table 4.

**Table 4:** Subcodes under facilitating conditions.

Code > Subcode	Number of items coded	Number of coding references	Percentage of participants that used
Facilitating Conditions	31	368	100%
> Account Management and User Interface (+/-)	24	114	77%
> Support (+)	22	73	71%
> dApps (+)	18	57	58%
> Support (-)	15	34	48%
> Technology Problems (-)	14	39	42%
> Crypto Management (+)	10	34	32%
> Crypto Management (-)	8	14	26%

First, while most participants mentioned the importance of **account management and user interface** during their interview, there was no clear agreement whether this factor supported or hindered the adoption and use of a BSM platform. For example, one participant said: “I jumped in there and quickly realized, okay, Hive, at the time it was still a bit rough around the edges. Still kind of is. The onboarding can be a bit of a challenge”. Here, the participant is describing features of the user interface as “rough around the edges,” meaning that it is not user-friendly and could use improvement. However, a different participant found the interface to support their adoption of the BSM platform in question. They described the account set-up process as straightforward: “The steps were very simple. The signup process, you just sign up [and] within [a] 24-hour period you get a notification for you to complete your account. Then you copy your password, and all that, the master password then, and that was it”.

A related subcode, **crypto management**, was used to note when participants discussed BSM affordances for managing cryptocurrency. Like the previous subcode, this one reveals positive and negative attitudes among the participants.

In relation to the subcode **support**, we also found a general disagreement regarding the support available when using BSM platforms. Even though 22 participants discussed how other community members offer help and support when technical questions arise, 15 participants expressed their dissatisfaction with the general lack of documentation and help resources from BSM platforms directly, as highlighted by this quote:

“The resources are few and far between. It was a good thing that I really like reading and I like exploring because it took searching around on different posts to find somebody that had posted about some problem and I went” oh okay that’s one problem”, and I’d read it through, and I would take some notes.”

The subcode **technology problems** was used exclusively in instances where the participant discussed issues related to the technical infrastructure of BSM platforms that hindered their adoption, or continued use, of the platform. This subcode is different from the ones discussed above as it was used for issues related to the inability to perform a specific task due to a failure in technology. Some technical problems mentioned were inherent features of blockchains, such as not being able to message someone privately or not being able to store media files on the blockchain, while most other references were made about unspecified bugs that were generally expected when using products in the early stages of their developments.

Nearly half of the participants in this group also raised the issue of slow or no internet access that affected their use, but was not caused by BSM; “So there was not much difficult, except maybe for Internet facility, sometimes it fluctuates…”

The subcode **dApps** reflected the overall positive sentiment towards the variety of decentralized applications available when using BSM. When this subcode was used, it was primarily used when the participants discussed how dApps supported their adoption or supported their current use of BSM platforms. The following quote captures this sentiment:

“Whereas with Hive, there’s a whole bunch of UIs [user interfaces]. For the Hive chain, you’ve got the active.fit, you’ve got PEAKD, you’ve got Ecency, you’ve got Hive.blog, you’ve got D.Buzz. You’ve got some beautiful, beautiful UIs coming out now [and] they’re all using the Hive chain. So, when you post on this particular URL, it’ll show up on chain in all of the other apps and—whether it be Android, iOS, Web TV, whatever, it will show it in the way that they want to see it.”

#### Financial incentives

As a distinct factor of BSM platforms is the potential for financial gains in the form of cryptocurrency, we used the code **financial incentives** for discussions related to this. Because this is a key aspect of BSM platforms, it is not surprising that this code was used by all participants (362 times). Three aspects underline this factor; we refer to them as content monetization, cryptocurrency volatility, and work opportunities (see Table 5).

**Table 5:** Subcodes under financial incentives.

Code > Subcode	Number of items coded	Number of coding references	Percentage of participants that used
Financial Incentives	31	362	100%
> Content Monetization	28	275	90%
> Cryptocurrency Volatility (+/-)	20	52	65%
>> Cryptocurrency Volatility (-)	9	19	29%
>> Cryptocurrency Volatility (+)	15	33	48%
> Work Opportunities	12	35	39%

The subcode **content monetization** was used where the participant’s adoption of BSM was influenced by their perceived ability to gain financially from using the platform. This was the most used subcode. As one of the participants explains it, one can earn tokens not just by sharing the original content but also by engaging with others:

“It was the money coming in that made me go ‘holy smokes man’, this could be huge for a lot of people who don’t even have an income. For example, if you comment on somebody else’s post, you can earn just as much, if not more, than the person who made the post, so that’s extremely powerful.”

In addition to being able to monetize content and engagement on BSM, another monetary aspect of using blockchains is the possibility of making money by investing in cryptocurrency. Twenty study participants mentioned this as critical to their consideration of adopting and using BSM (coded as **cryptocurrency volatility**). This was one of the subcodes with differences in opinion since not everyone referencing this aspect viewed it as a benefit; some also expressed concerns that the volatility of the crypto market could result in a loss of their funds.

Finally, the subcode **work opportunities** was used in instances where the participant discussed the utility of BSM for creating work and career opportunities. Some participants discussed their desires for their activity on BSM platforms to turn into work; with one participant saying: “And I’m thinking of making this blogging a full-time job”. Other participants shared personal stories or stories of others they knew who turned their use of BSM into work. For example, one participant shared the story of how their friend opened a makeup store using their earnings from Hive.

#### Effort expectancy

The code **effort expectancy** was used 326 times by nearly all participants (n = 30) when discussing the degree to which the BSM platform is perceived to be easy or difficult to operate. These instances often involved participants mentioning the “learning curve” they had to navigate as new users of BSM (see Table 6).

**Table 6:** Subcodes under effort expectancy.

Code > Subcode	Number of items coded	Number of coding references	Percentage of participants that used
Effort Expectancy	30	326	97%
> Needed Knowledge	28	131	90%
> Time Needed	25	97	81%
> Learning Community Norms	18	45	58%

Three subcodes were used to specify different aspects that the participant found to be particularly easy or difficult. First, the subcode **needed knowledge** was used when the user communicated needing understanding of certain topics to use BSM platforms. Most participants who raised this issue expressed difficulties when starting to use a particular BSM platform. The only exception were participants with some prior technical experience or knowledge of how blockchain technologies work. For example, one participant stated that because they had previous knowledge about general technology use, adopting BSM was not difficult for them. However, the implication here is that if someone did not have this general knowledge of technology, adoption would be difficult.

The second most used subcode was **time needed**. It was used when the participant indicated they required time to learn how to use BSM. For example, one participant stated: “Well, yes. For instance, to write a good post, to write your review, it will take some time. You have to do your research, so it will take some time”, indicating that time is needed to make a good post on the platform.

The third subcode **learning community norms** was used when the participant communicated that they needed to understand the community norms of a BSM platform to use it seamlessly. For example, one participant reflected: “Needing to know the correct tags for things, and now there’s communities and tags, but back at the time, there were no communities; it was a large learning curve.” This participant is discussing how they had to learn to correctly tag their posts, which is an unsaid norm rather than related to the technological aspects of using the platform.

### Performance expectancy

**Performance expectancy** was operationalized as instances in which participants reported an increased perception of the usefulness and efficacy of BSM for specific tasks. As shown in Table 7, this code was applied 221 times across 27 interviews.

**Table 7:** Subcodes under performance expectancy.

Code > Subcode	Number of items coded	Number of coding references	Percentage of participants that used
Performance Expectancy	27	221	87%
> Blockchain vs. Mainstream	23	83	74%
> Blockchain vs. Blockchain	17	89	55%
> Targeting a Niche Audience (+/-)	14	39	45%
>> Targeting a Niche Audience (-)	10	28	32%
>> Targeting a Niche Audience (+)	5	11	16%

The first subcode **blockchain vs. mainstream** was used when participants discussed BSM’s advantages, particularly for engaging other users, compared to MSM platforms. For example, one participant said: “I see that Hive [is] a better place one can spend most of their time instead of whining away on Facebook and WhatsApp”.

Participants not only compared their experiences of using BSM to MSM platforms but also provided evaluations and comparisons of their experiences across various BSM platforms or within dApps of the same BSM platform. These instances were categorized under the subcode **blockchain vs. blockchain**. Such sentiments were shared by approximately half of the participants, usually those with more experience in using BSM platforms. One of the main differentiating factors among BSM platforms identified under this subcode is the presence or absence of transaction fees for operations on the blockchain. In this regard, Hive was frequently cited as a preferred platform by the participants, as it does not impose transaction fees.

We also found that the ability of a BSM platform to attract the development of various third-party applications was identified as a key factor in determining which BSM platform to choose from the multiple options available. One participant explained their preference for Hive over other BSM platforms such as Minds, Bastyon, and Float, which they had previously experimented with, by citing the strong developer community and the abundance of third-party applications built on top of the Hive blockchain as key reasons for their preference.

The final subcode identified in relation to performance expectancy pertained to the capability of the BSM platform to target a specific demographic. This aspect was coded as **targeting a niche audience**. The analysis revealed a mixed perspective among participants, with some highlighting the ability of BSM to reach a specific audience as an advantage. In contrast, others viewed it as a barrier to the adoption and sustained usage of the technology. Specifically, the former group viewed the smaller user base of BSM platforms as an advantage, as it allows for a more targeted audience and increased interest in niche topics. On the other hand, the latter group argued that the limited user base of BSM platforms hindered the effectiveness of disseminating their content to a broader audience, as exemplified by the following quote:

“… if you want to reach the largest possible audience, you will not get it on Hive because it’s too small, it’s not trending yet. Maybe it will become one day, but at the moment, I don’t think it is where you can get the largest possible audience.”

#### Risk

The concept of **risk** was used as a code to classify statements about the perceived negative consequences associated with using BSM. This code was applied 168 times across 27 interviews. Within this code, various subcodes were created to distinguish the different types of risks mentioned by participants (see Table 8). Notably, most of the risk factors identified in the interview data were prevalent across both MSM and BSM.

**Table 8:** Subcodes under risk.

Code > Subcode	Number of items coded	Number of coding references	Percentage of participants that used
Risk	27	168	87%
> Power Dynamics	14	42	45%
> Anti-Social	14	31	45%
> Security and Privacy	12	29	39%
> Platform Longevity	11	22	35%
> Scams	8	26	26%
> Bots	4	7	13%
> Legal	3	6	10%
> Environmental	3	5	10%

The subcode **power dynamics** was referenced concerning situations where ’whales,’ users holding a significant stake exerted negative influence or had the potential to do so on the platform’s operations or other users. This is because whales possess not only the capability to manipulate the token’s price through buying or selling large quantities but also the potential to shape the platform’s content through the support or opposition of specific content creators. As one participant explained:

“If you get [a whale] that decides to, what we would refer to as, go rogue, and they’ve decided that they’re going to set the tone as to who can do what on the platform and they start downvoting people, it can create chaos and it can hurt a lot of smaller [creators].”

The subcode **power dynamics** also emerged in discussions regarding the creation of Hive as a community-driven fork (clone) of Steemit following the acquisition of Steemit by the Tron Foundation. This was perceived as a precautionary measure, as the acquisition concentrated a substantial number of tokens under the control of a single entity, raising concerns about the potential for undue influence on the platform’s future direction [61].

Another risk factor associated with the use of BSM is the potential for encountering anti-social behavior, such as hostility, conflicts, and bullying. The subcode **anti-social** was employed as a broad term to encompass a wide range of behaviors within this category. For instance, during one of the interviews, a participant recounted an online altercation with other users, acknowledging that such incidents are to be expected in any “social” space:

“… because this is social, sometimes there can be drama or—I’ve tried to always have a good relationship with everyone, but sometimes it has been inevitable. So, I have had fights, or drama, or because this is social, people assume things. Or well one time there was a group of people abusing, right away abusing, and I have to call them out, and it was a drama. But that is not something a regular user will experience. I did that because I’m a community leader …”

The next subcode, **security and privacy**, was used for discussions of the potential risks to users’ security and privacy on the platform. For example, due to the publicly accessible nature of all transactions on the blockchain, several participants expressed worries about sensitive information being readily accessible to anyone. In some other instances, the concern was related to the immutability of the content posted on the blockchain, which cannot be altered or removed, as explained by one participant: “…I have not shared my last name, I have not shared photographs of me, or exactly where I live…So I also do not share anything because I know that it cannot be erased and that it will stay there forever.”

The subcode **platform longevity** was used to capture participants’ worries about the potential shutdown of BSM platforms, which could result in loss of data and earned cryptocurrency, as well as the wasted efforts invested in building an online reputation. For instance, one participant highlighted a common risk in BSM platforms—the rapid turnover rate of smaller platforms:

“One of the challenges I am envisioning is the issue of continuity, because from the track record, most of these blockchain platforms, some of them do not last more than 10 years, some of them five years, some of them six. So, because of that it creates a little fear, like what will become of me?”

The subcode **scams** was employed when participants discussed the presence or perceived presence of scams, phishing, or fraud on BSM platforms. This subcode was recorded 26 times in 8 interviews. For instance, one participant relayed an experience of a family member being defrauded through BSM:

“There are so many social media platforms [where] people have been duped. Some of them will ask you to put in your money and before you know it, it becomes stolen. Like my daughter was also duped; she engaged herself in one of them and she lost her money. So, it made me not even want to hear about some of these blockchain social media.”

Despite this negative experience, neither this participant nor others who raised this issue considered it a sufficient deterrent to discontinue the use of BSM altogether. Instead, they emphasized greater caution when connecting with others on BSM, particularly in financial transactions.

The subcode **bots** was applied when participants mentioned the actual or perceived presence of malicious bots or automated accounts on the platform. Only 4 participants brought up bots as a potential risk. One participant expressed worry about bots that vote down posts and the absence of a way to block such automated accounts. Another participant shared their mixed experiences on a BSM platform called read.cash, raising questions about the transparency of the AI bot that automatically selects posts for promotion:

“You have the one disadvantage of read.cash is that you earn mainly from the bots that [are] overseeing everything on the platform. So, at their own discretion, they gift to you what they feel you should earn. So, it’s not like on Hive, that other writers [and] other members can tip. So, when they tip you, you actually accumulate a certain amount. On read.cash, they have a bot, and it’s their own discretion. Being good is relative. So, you may think your article is good, [but] you don’t get a tip, you don’t get paid. So maybe once or twice you might be disappointed on that service.”

The subcode **legal** was applied when 3 participants referenced the risks posed by government regulations. Specifically, these participants expressed concerns about either potential government regulations or the absence of regulations. As one participant explained: “You can’t sue Hive for anything because who are you going to sue? We don’t have a CEO or anyone that represents us, so who are you going to sue? You’re going to sue the person.”

Even though environmental concerns (specifically the high energy consumption and resulting carbon footprint associated with blockchain mining) is a recurrent critique of the technology [62], our participants did not frequently discuss this issue. The subcode **environmental** was only used in 3 interviews. One example was a conversation in which a participant discussed alternative types of blockchains based on so-called Proof-of-Stake that do not necessitate coin mining as a means of validation.

#### Social influence

The influence of friends, family, and online influencers was a significant factor in some participants’ decision to adopt BSM. This factor was coded as **social influence** and cited 123 times by 27 participants (see Table 9).

**Table 9:** Subcodes under social influence.

Code > Subcode	Number of items coded	Number of coding references	Percentage of participants that used
Social Influence	27	123	87%
> Personal Circle	18	79	58%
> Influencer	6	20	19%
> Media	6	14	19%
> Industry Trend	5	8	16%

The subcode **personal circle** was used to record instances in which the adoption of BSM was influenced by recommendations from someone within the user’s social network. This was the most common form of social influence. This could be an online or offline friend, a family member, or a co-worker.

The subcode **influencer** was used to capture instances in which the use of a BSM platform was motivated by exposure to a post on a MSM platform, usually from an individual with a substantial following. For instance, one participant recounted discovering BSM through a post on Reddit: “[…] I read some guy talk about this new technology, a social media blockchain that allows you to earn a cryptocurrency by publishing content. So, it got me really curious.”

The subcode **media** was used to record instances in which the adoption of a BSM platform was motivated by exposure to online content or websites from formal or informal organizations. As one participant explained: “I stumbled across it [online], and I do have to say stumbled across because I was just roaming around one day; I think I was down a rabbit hole. Anyways, I came across Steem.”

The subcode with the least usage in this category was **industry trend**. It was only used in 5 interviews to capture instances in which the adoption of a BSM platform was driven by technology companies or general industry trends without referencing a specific entity. For example, one participant explained that while they knew about blockchain and cryptocurrency since around 2014, they only got into it a couple of years later when “everybody was hearing about this Bitcoin, and how it went from 1000 to 20000.”

#### Habit

Another key factor in adopting and using a BSM platform was the extent to which its usage had become routine. Instances in which participants described their use of a BSM platform as routine or regular were coded as a **habit** 105 times in 27 interviews. There were no subcodes in this category.

This code was also applied when participants discussed having a history on the platform or experience with other blockchain platforms. For example, one participant recounted their history on the Steemit platform before moving to HIVE: “Well I’m right now using the Hive blockchain for about two years. But I was on a Steemit platform for about four years. I was on a Steemit from 2018. And that’s the first moment when I started to use these blockchain platforms.”

#### Content moderation

BSM platforms lack a central governing body, resulting in a lack of moderation of content compared to MSM platforms. We used the code **content moderation** to capture discussions regarding content moderation (or the lack thereof) on BSM platforms. It was mentioned 248 times by 24 participants. Our analysis identified 5 distinct subcodes related to content moderation, as shown in Table 10.

**Table 10:** Subcodes under content moderation.

Code > Subcode	Number of items coded	Number of coding references	Percentage of participants that used
Content Moderation	24	248	77%
> Freedom of Speech	17	71	55%
> Ownership	14	68	45%
> Immutability (+/-)	14	27	45%
> Open Access	13	44	42%
> Content Discovery	8	18	26%

The subcode **freedom of speech** was applied when participants stated that their adoption or use of BSM was influenced by their belief in the ability to express their opinions without interference from the platform. The following quote is representative of the type of discussions that were coded using this subcode:

“And essentially you can’t be canceled. So, I could say something that one side of an aisle or the other doesn’t find favorable and they don’t have any means to be able to go, ‘you know what, you need to take that down’. It’s like well, I don’t have to take it down…”

The subcode **ownership** was used to record instances in which participants felt they had greater control and ownership over their account, content, or data, mainly due to the decentralized nature of BSM. This aspect of BSM was often discussed in the context of having control over one’s digital keys to access various dApps or crypto exchanges, as illustrated by the following quote:

“And Hive, like Bastyon too, you own the keys, like your private keys, you own them, [and] that means you own your cryptocurrency. On any of the exchanges, foreign banks, or anything where you don’t have your keys, you don’t own your money. On Hive you do.”

The subcode **immutability** was used to reflect discussions about the characteristics of blockchains that content cannot be removed. This was the only subcode in the category showing a mixture of positive and negative views. Some participants appreciated the permanence of their data on the blockchain, while others worried about the potential for sensitive information to remain there indefinitely.

The subcode **open access** was used to capture instances in which participants discussed BSM platforms being open in all aspects: built with open-source tools, freely accessible to anyone, and content is copyright-free for anyone to use. For example, one participant stated that anyone with the skills could build anything on the Hive blockchain: “On Web 3, like on Hive, if you want to build a front end or you want to build an app, or you want to do something on Hive, you don’t need permission. All you need to do is know how to program and tie into the chain and you’re good to go”.

The subcode **content discovery** was used to record discussions about how content is discovered on BSM platforms. The lack of algorithmic filtering was often cited as a benefit in finding content on BSM, as captured by the following quote: “​​When you’re on Web 2 you’re getting algorithms that are going to serve you up content and contact with people that are based on things that you’ve shown an interest in. On Web 3, and particularly on social media sites like Hive, there is no algorithm there so you can find yourself in contact with people literally all over the world”.

#### Trust

As previously noted in the paper, trust plays a crucial role in technology adoption, including BSM platforms. Thus, the **trust** code was established to capture discussions related to trust and was used 134 times by 22 participants. The code consists of three subcodes, which are displayed in Table 11.

**Table 11:** Subcodes under trust.

Code > Subcode	Number of items coded	Number of coding references	Percentage of participants that used
Trust	22	134	71%
> Trust in BSM	21	51	68%
> Lack of trust in MSM	15	43	48%
> Transparency	8	35	26%

The subcode **trust in BSM** was used when participants indicated that their perceived trust in the platform, developers, or users influenced their adoption and use. This typically involved instances in which the user believes that the witnesses of the platform or other users have their best interest in mind. For example:

“I think that in this case, Hive can make you believe. Hive can make you trust the people in the community, even if they don’t know you, you could trust it but from looking at all the testimonies, looking at the things that people say using the hashtag Hive on Twitter. If people just look a little bit on the Hive hashtag on Twitter, I think that people could trust these platforms, without even making some questions.”

In contrast, the subcode **lack of trust in MSM** was used when participants discussed their lack of trust in MSM. This included discussions of how MSM platforms do not have their best interest in mind. For example, one participant mentioned how platforms like Facebook misused user data in the past, stating that MSM platforms are “very exploitative—very, very.”

Finally, the **transparency** subcode was used to record instances in which participants discussed the positive aspects of transparency on the platform. It was necessary to differentiate between positive and negative sentiments because participants also raised concerns about transparency being a risk. These instances were coded as **security and privacy** under the **risk** code. The following quote is an example of a participant discussing the anti-corruption benefits of the transparency offered by BSM:

“But the thing is with that transparency that corruption becomes way more difficult. You know if I’m paying this plumber for a service and he says ‘no, no, you never paid me’. Well here, look at the chain, here’s my username, their username. There is a transaction right there, there’s no hidden addresses or hidden balances or anything like that.”

## Discussion of research questions

Building on the coding results, we will address the research questions below.

### RQ 1: What motivated early adopters to use blockchain-based social media platforms?

Social influence played a pivotal role in the adoption of BSM. Several early adopters stated that they learned about BSM and started using them due to recommendations from individuals in their personal networks. This trend is likely driven by the fact that most BSM users we interviewed demonstrated a keen awareness of their role as stakeholders in their respective communities and recognized the significance of actively promoting and recruiting new members. As a result, they frequently reach out to friends and family and cross-post content to other social media platforms.

Financial incentives are cited as another driving factor for adopting the technology. Most users were attracted to BSM platforms because of the potential to earn reward tokens in the form of cryptocurrency. For instance, some interviewees from developing countries, such as Nigeria, reported that monetized BSM platforms offer a means of making a living and even financially supporting community projects.

The third critical factor influencing slightly over half of users’ decision to adopt BSM is the ability to express opinions without interference. Those who joined BSM for this reason valued specific inherent features of blockchain technology, such as the permanence of records and the open-source and accessible nature of BSM. This group also appreciated the lack of algorithmic filtering for content discovery on BSM. However, some individuals in this group acknowledged that a certain level of automation may become necessary to curate user content if and when BSM become more widely adopted.

### RQ 2: What factors contribute to the continued use of blockchain-based social media platforms?

The most frequently cited factor that led users to continue using their preferred BSM platform was hedonic motivation. All participants said that they enjoyed using the platform and interacting with other users. When describing their experiences, most mentioned their feelings of belonging, recognition, and the importance of sustained membership. Additionally, they valued the ability to share and learn from others about their areas of expertise.

Although the support for online communities is not unique to BSM, the emergence of distinct communities differentiates them from other social media platforms. Many communities mentioned by participants could be categorized as communities of practice. In these communities, members work together to enhance their online research skills and produce ‘high-quality’ content that would attract maximum engagement. Additionally, we found that the presence of cryptocurrency behind social media content often instilled a sense of collective empowerment among community members on a BSM platform. Some users sought to leverage this financial resource to support local causes that the online community had selected through a vote. Moreover, there appeared to be a stronger connection between users and BSM developers. This contrasts with centralized platforms, where a user’s interaction with developers is typically limited to filling out a one-way feedback form.

Habit and prior experience with different BSM platforms also positively impacted participants’ decision to continue their use of BSM. Participants who enjoyed BSM interfaces and their functionality in managing user accounts were more likely to continue using the platform. Still, their experiences varied across platforms and dApps used and when they first started using them. These differences reflect the rapid advancements in the BSM world, with various development projects at different stages and users with varying technical skills.

### RQ 3: What are the main barriers to adopting and using blockchain-based social media platforms?

Participants in our study identified the main barriers to adopting BSM as the effort required to create an account and learn how to use and be effective on these platforms. While these onboarding-related barriers have not prevented our study participants from using BSM, most acknowledged that it could be a challenge for those without prior technical experience or knowledge of blockchain technologies. Furthermore, users without personal support networks, such as friends who could guide them through the sign-up and onboarding process, may also find it challenging to use these platforms.

In terms of interface affordance, some have flagged the limitation of blockchain in hosting private direct chats as a hindrance to socializing with others. This limitation led to the need to use other communication channels to stay in touch with members of the community outside the blogging activities they are typically engaged in when using BSM.

Risks such as the potential for financial scams, privacy and security breaches, and the possibility of a BSM platform going out of business, were also identified as barriers to the adoption and use of the technology.

### RQ 4: How does using cryptocurrencies as incentives for content creation and engagement affect the behavior and perception of social media usage?

For most participants, earning reward tokens was the main attraction of using BSM platforms. However, some also found earning enough rewards and improving engagement on their posts was challenging. Those primarily driven by financial incentives also preferred platforms with low or no transaction fees, such as Hive. This emphasizes the need to consider the economic model of a BSM platform when assessing its potential for adoption and use.

Moreover, the discussion of monetizing content on BSM uncovered a conflict between ‘whales’ (users who are financially invested in the platform) and the rest of the community. Although BSM infrastructure is decentralized, a certain level of hierarchy exists in the governance structure; nearly half of the study participants raised the issue of power dynamics between ‘haves’ and ‘have-nots’. This includes the reliance on whales for upvotes, since they can influence the platform’s content by supporting or opposing specific content creators. While whales can provide liquidity and stability, a proper governance mechanism and equitable token distribution are critical to minimize negative impacts caused by a few prominent stakeholders controlling the platform and influencing its content.

### RQ 5: How has adopting blockchain-based social media platforms influenced individuals’ usage habits and behaviors on mainstream social media platforms?

Most study participants reported having limited interaction with MSM. Those who still used MSM indicated that they did so infrequently, primarily to connect with family or friends who were not yet using BSM. In some cases, BSM users also used MSM to facilitate engagement with other BSM users since direct messaging is not supported on BSM platforms. Finally, some BSM users had to rely on MSM platforms to cross-promote their content published on BSM to attract a larger viewership. From their perspective, the user base on BSM is still very niche, hindering the ability to disseminate their content widely. This finding suggests that BSM remains a niche product and that these platforms are only able to meet some of the use and gratification needs of current social media users.

## Conclusions and future work

If the process of joining BSM platforms becomes smoother and their interfaces become more approachable, we could see an increase in their user base, mainly because users can monetize their content and engagement. However, our results indicate that the absence of content moderation, security, and privacy poses significant challenges and hinders the widespread adoption of BSM. This presents a paradox, as similar issues prompted some users to abandon MSM such as Facebook and YouTube.

From the theoretical side, the results confirmed that the UTAUT model and its common extensions effectively predict the main factors that influence the adoption and use of BSM. Hedonic motivation was the most frequently cited factor, with all participants enjoying the platform and interaction with the community. Facilitating conditions were also frequently mentioned but reflected both positive and negative experiences with the technology. Effort expectancy was cited as a barrier to adoption due to the increased effort required. In contrast, performance expectancy was seen as a positive aspect, with users perceiving BSM as more useful and efficient than MSM for specific tasks. Social influence and habit played a role in the initial decision and continued use of BSM platforms, respectively. Risk and trust were also frequently discussed, but participants still expressed trust in the technology despite the associated risks.

The study identified additional factors that influence the adoption and use of BSM platforms, which are not currently included in the UTAUT model as reported in the literature. These factors are financial incentives and content moderation. Financial incentives, such as the opportunity to earn cryptocurrency, were identified by all participants as a significant driver for their decision to join a BSM platform. The integration with cryptocurrency sets BSM apart from MSM and offers a new way for users to monetize their content and engagement. Content moderation was also a key factor, particularly for those dissatisfied with the moderation policies of MSM.

While established social media giants like Facebook and WeChat remain prominent, BSM platforms are potential disruptors with millions of users and investments. Future studies should further explore the potential and challenges of these platforms to advance our understanding of their impact on society. For example, due to the novelty of these platforms, it is unclear if and how trust and safety standards commonly developed and enforced by MSM would apply in blockchain networks since no single entity is tasked with data stewardship. Future research should also investigate to what extent demographic characteristics, such as gender, age, income level, and social status, moderate the adoption and use factors identified in this study. Finally, future work could also reveal how global legislative efforts on content moderation, cyberbullying, and micro-targeting affect the operation and adoption of these decentralized platforms.

## Supporting information

S1 Appendix Interview open-ended questions. (DOCX)

S2 Appendix Codebook. (DOCX)
