# Supplementary material for: The lure of decentralized social media: Extending the UTAUT model for understanding users’ adoption of blockchain-based social media
Source: PLoS One. 2024 Aug 7;19(8):e0308458. doi: 10.1371/journal.pone.0308458 (PMC11305580; doi:10.1371/journal.pone.0308458)
Supplement: S1 Appendix — (DOCX) [file pone.0308458.s001.docx]

**S1 Appendix. Interview Open-Ended Questions**

Introduction

- Which blockchain-based social media platforms do you currently use?
  - If more than one: Which one do you use most often? [For rest of interview, ask them about this platform specifically]

Initial use

- How did you first find out about blockchain-based social media?
- When did you first initially sign up for [platform]?
- Why did you choose to sign up?
- How easy or difficult was it for you to learn how to use [platform] when you first started?

Current use

- Thinking about your use of [platform] now, what do you use it for?
- Do you find there are any benefits to using [platform]? If yes, what are they?
- Do you find there are any challenges to using [platform]? If yes, what are they?
- Do any of your peers use [platform]? How does that influence your use of [platform], if at all?

Mainstream social media platforms

- Now, I would like you to think about mainstream social media platforms, like Twitter, Reddit, Facebook, and Instagram. Do you currently use these types of platforms, or have you used them in the past?
  - If currently using - What is the division of your time? For example, do you spend 50% of your online social media time on traditional platforms, and the other 50% on blockchain-based platforms?
- Has your use of blockchain-based social media platforms changed your use of mainstream social media platforms in any way?
  - If yes, how?
  - If no, why not?
- We already talked about the benefits of blockchain-based social media platforms. I would now like to ask you about this in relation to traditional platforms.
- Do you think there are benefits of using blockchain-based social media platforms compared to traditional?
