# Supplementary material for: The lure of decentralized social media: Extending the UTAUT model for understanding users’ adoption of blockchain-based social media
Source: PLoS One. 2024 Aug 7;19(8):e0308458. doi: 10.1371/journal.pone.0308458 (PMC11305580; doi:10.1371/journal.pone.0308458)
Supplement: S2 Appendix — (DOCX) [file pone.0308458.s002.docx]

**S2 Appendix. Codebook**

| Name | Description |
| --- | --- |
| **01 - Performance Expectancy** | The degree to which the blockchain-based social media (BSM) platform is perceived as helpful to the user. The participant discussion may include the following:   1. The usefulness of the BSM platform to them for their goals or work/job, 2. The BSM platform increases the user’s efficiency at some relevant tasks, 3. That they have some extrinsic motivation (e.g., receiving or expecting to receive some external reward) for using the BSM platform, 4. The BSM platform offers them a relative advantage compared to people who do not use the platform, 5. The BSM platform leads to personal development in terms of skills. |
| Blockchain vs. Blockchain | Instances where the BSM platform is perceived to have an advantage over another BSM platform. |
| Blockchain vs. Mainstream | Instances where the BSM platform is perceived to have an advantage over mainstream platforms. |
| Reaching audience (-) | Instances where a user perceives that the audience on the BSM platform is not large enough to reach their desired audience. This includes discussions of needing to market their content on traditional platforms to reach a wider audience. |
| Reaching audience (+) | Instances where users perceive that they can reach a wider audience (for their content) through BSM platforms. |
| **02 - Effort Expectancy** | The degree to which the BSM platform is perceived to be easy (or difficult) to use.  The user may mention things like: (1) the BSM platform was/is easy to use/learn to use, or easier to use/learn to use than they expected, or (2) the BSM platform was difficult to use or more challenging to use than they expected. |
| Needed Knowledge | Users are expected to know about specific topics to use the BSM platform. The perceived ease of use is high if the user has the knowledge or low if the user does not have the knowledge. |
| Learning Community Norms | New users must learn the norms of the BSM platform to use it seamlessly. This may include learning which tags to use, how to write “good” posts, or which communities to post in. |
| Time needed | Instances where the user mentions that time is needed to learn how to use the BSM platform or build your network or time is necessary to talk about the platform/promote it outside the network because “there are no CEOs to do it.” |
| **03 - Social Influence** | The degree to which using the BSM platform is approved of or endorsed by the social network of the individual or influential others. |
| Industry trend | When the adoption was driven in part because of action(s) by an influential technology company or other organizations. |
| Influencer | The user adopted BSM because they were exposed to a post from an individual rather than a formal or informal organization/publication, regardless of the individual’s number of followers. |
| Media | The user adopted BSM because they were exposed to something online from a formal or informal organization/publication. |
| Personal circle | The user adopted BSM because they heard about them from someone in their personal circle or social network, including friends (online/offline), family members, co-workers, neighbors, etc. |
| **04 - Facilitating Conditions** | The degree to which an individual believes that organizational and technical infrastructure exists to support the use of the BSM platform. |
| Account management and UI | The user’s adoption of the BSM platform was related to systemic issues on the platform pertaining to accounts (e.g., setting up accounts and managing keys) or the overall usability of the user interface. This also includes instances where the user discusses the need for developers to automate, optimize, streamline (etc.) a process relating to the BSM platform rather than stating that platform users need knowledge. |
| Crypto Management (-) | The technical infrastructure of the BSM platform hinders users’ management of cryptocurrency. For example, the exchange of tokens is difficult because of the platform's features. |
| Crypto Management (+) | The technical infrastructure of the BSM platform supports users’ management of cryptocurrency. For example, users may mention low transaction/gas fees or quick transaction speeds. |
| dApps | Instances where the user discusses the variety of dApps (decentralized applications), user interfaces (UIs), or front ends that there is to choose from when it comes to BSM platforms. |
| Support (-) | Tangible resources, such as documentation, tutorials, guides, etc., were/are not available to support the use and adoption of the BSM platform. |
| Support (+) | Tangible resources, such as documentation, tutorials, guides, etc., were/are available to support the use and adoption of the BSM platform. |
| Technology Problems | Problems that occur either during the sign-up process or during the usage of the BSM platform that hinder the adoption or continuing use of BSM. For example, poor internet connection, login difficulties, platform crashes or the platform becomes unavailable. |
| **05 - Hedonic Motivation** | The degree to which different aspects of the BSM platform is perceived to be enjoyable. |
| Growing the Community | Instances where the user discusses the desire to use online or offline channels to get more people using BSM platforms. |
| Crowdfunding and Charity | The user’s adoption of the BSM is influenced by their perceived ability to initiate or contribute to crowdfunding, crowdsourcing, citizen science, or charity opportunities through the BSM platform. The user perceives this to be enjoyable not for their direct benefit but for the benefit of others or “the greater good.” |
| Learning and Teaching | The use of the BSM platform is perceived to be fun or enjoyable because the user can learn from others or teach others. |
| Novelty Enjoyment | The use of the BSM platform is perceived as fun or enjoyable by the user because it is perceived as unique, interesting, or novel. |
| Sense of Community | The use of the BSM platform is perceived to be fun or enjoyable by the user because of the community aspect; making friends or meaningful connections or expanding their social network is seen as a key benefit of the technology. |
| Usage Enjoyment | The use of the BSM platform, in general, is perceived to be fun or enjoyable by the user. |
| **07 - Habit** | The extent to which usage of the BSM platform has become a routine. This also includes instances where the user discusses having a history on the platform (e.g., being a long-time user, referencing using Steemit previously). |
| **08 - Financial Incentives** | Use of the BSM platform is related to financial or monetary gain. |
| Content Monetization | The user’s use or adoption of the BSM platform is influenced by their perceived ability to gain financially from creating and posting content on the platform or other activities (online or offline). |
| Cryptocurrency Volatility (-) | The user believes that using the platform may lead to a loss and that this loss will result from the volatile cryptocurrency market. |
| Cryptocurrency Volatility (+) | The user’s use or adoption of the BSM platform is supported by the volatility of the cryptocurrency market; users see the volatility as a benefit and an opportunity to earn funds. |
| Work opportunities | The BSM platform is perceived to be beneficial because it offers users work opportunities that they did not previously have access to. This could be because of work on the platform or learning skills that have/could lead to additional work opportunities. |
| **09 - Trust** | The belief that others (see below for examples) will fulfill their obligations to the user. |
| Trust in Blockchain Social Media Platforms | The use of the BSM platform is influenced by users’ perceived trust in the platform or its users; the user believes that the witnesses of the platform or other users of the platform have their best interest in mind. |
| Trust in Mainstream Social Media Platforms | The use of the BSM platform is influenced by users’ perceived lack of trust in mainstream social media platforms or their users; the user does not believe that the owners of traditional platforms or other users of traditional platforms have their best interest in mind. |
| Transparency | Instances where the user discusses transparency on the platform in a positive light, for example, discussion of “trustless transactions” or their ability to verify transactions because everything is available on the blockchain. |
| **10 - Risk** | The degree to which the user perceives that the technology will lead them to experience some disadvantage. |
| Platform Longevity | The user believes that the use of the platform may lead to a loss and that this loss may result from prematurely shutting down. |
| Bots | Instances where the user discusses the existence or perceived existence of bots or automated accounts on the platform. This is seen as a risk or disadvantage of using BSM platforms. |
| Legal | References to policies, regulations, and laws concerning blockchain technology (including tax-related implications). |
| Environmental | Instances where the user discusses the environmental implications of coin mining. |
| Security and Privacy | Discussions of how the existing technical infrastructure of the BSM platform relates to users’ views on security and confidentiality. The user may feel like the existing technical infrastructure of the BSM platform supports or hinders (1) the security of the platform, (2) how secure they feel on the platform, or (3) their ability to keep their personal information on the platform confidential. |
| Scams | Instances where the user discusses the existence or perceived existence of scams, phishing, fraud, etc., on BSM platforms. |
| Anti-Social (-) | Instances where the user discusses the possibility of encountering anti-social behavior (e.g., hostility, fights, conflict, bullying, drama) on the BSM platform as a risk. |
| Power Dynamics | References to instances when a blockchain user with a large stake in the platform (‘whale’) negatively influences its operations. Another scenario is when a person or a company (investor) buys a large stake in a BSM platform. |
| **11 - Content Moderation** | Includes any discussion of censorship or shadow-banning (or lack thereof) on the platform. |
| Freedom of Speech | The use of the technology is influenced by users’ perception that they can speak freely without the platform's intervention. |
| Content Discovery | Discussions of content consumption on a blockchain platform, how content is discovered and the role of algorithmic filtering. |
| Ownership | Instances where the user references the ability on BSM platforms to own your account, content, data, etc. |
| Immutability | Instances where the user discusses the inability to remove or delete content/data from the blockchain. This includes discussions of this feature in a positive or negative light. |
| Open access | References to the BSM platform are open in all aspects; it is built using open-source tools, access is open to anyone, and the content is copyright-free for anyone to use. |
